# Europe-wide maps of biomass density based on satellite remote sensing data for 2017, 2020, 2021 and 2023

**DOI:** 10.1016/j.dib.2026.112536

**Published:** 2026-02-02

**Authors:** Maurizio Santoro, Oliver Cartus, Arnan Araza, Martin Herold, Jukka Miettinen, Ake Rosenqvist, Kazufumi Kobayashi, Takeo Tadono, Frank Martin Seifert

**Affiliations:** a Gamma Remote Sensing, Worbstrasse 225, 3073 Gümligen, Switzerland; b Earth System and Global Change, Wageningen University & Research, Droevendaalsesteeg 3a, 6708 PB Wageningen, the Netherlands; c Helmholtz GFZ German Research Centre for Geosciences, Remote Sensing and Geoinformatics Section, Telegrafenberg, Potsdam, Germany; d VTT Technical Research Centre of Finland, P.O. Box 1000, 02044 Espoo, Finland; e solo Earth Observation, Kachidoki 6-3-2, Chuo-ku, Tokyo 104-0054, Japan; f Remote Sensing Technology Center of Japan, Tokyu Reit Toranomon Bldg 3F, 3-17-1 Toranomon, Minato-ku, Tokyo, 105-0001, Japan Tokyo, Japan; g Japan Aerospace Exploration Agency, Earth Observation Research Center, Tsukuba-shi, Ibaraki-ken, 305-8505, Japan; h European Space Research Institute, European Space Agency, 00044 Frascati, Italy

**Keywords:** Forest, Sentinel-1, ALOS-2 PALSAR-2, LiDAR, GSV, AGB, BGB

## Abstract

Spatially explicit information on forest structure and biomass is needed to meet the monitoring and reporting requirements of several European policies. Satellite images enable mapping and monitoring of the Europe’s forest resources through operational observations from the Sentinel-1 Synthetic Aperture Radar (SAR) and the Advanced Land Observing Satellite 2 (ALOS-2) Phased Array l-band SAR 2 (PALSAR-2) instruments. Data acquired in 2017, 2020, 2021 and 2023 were used to generate annual maps of forest biomass variables, namely Growing Stock Volume (GSV), Aboveground Biomass (AGB) and Belowground Biomass (BGB), with a pixel size of 20 *m* × 20 m. All products are in the geometry of the Sentinel-2 tiling system. A spatially averaged map with a pixel size of 100 *m* × 100 m (1 hectare) in geographic projection is also supplied, for users who do not require the highest spatial resolution. The maps were generated with a fully documented processing chain that includes (i) pre-processing of the SAR data to create stacks of co-registered terrain geocoded images of the backscattered intensity and (ii) inversion of a physically-based model to estimate GSV. AGB and BGB were subsequently estimated using allometric relationships. Per-pixel standard deviations were computed for each biomass variable by propagating uncertainties from both the SAR observations and the model parameters. The maps clearly reproduce the expected spatial patterns of forest biomass across Europe and provide sufficient spatial detail to identify biomass dynamics related to, e.g., logging and regrowth. Validation against measurements collected by National Forest Inventories (NFIs) indicates poor agreement with map values at the pixel scale, with errors larger than 50% of the reference biomass. The correspondence substantially improved for spatial aggregates, such as administrative units, for which the bias was mostly negligible and the mean square error was below 30% of the reference value. The number of ALOS-2 PALSAR-2 images affected the inter-annual consistency of the maps, which was lower in regions with only one or two observations per year.

Specifications Table

**Table:** 

Subject	Earth & Environmental Sciences
Specific subject area	Pan-European maps of Growing Stock Volume (GSV), Aboveground Biomass (AGB), and Belowground Biomass (BGB) in woody vegetation with a pixel size of 20 *m* × 20 m for the years 2017, 2020, 2021 and 2023. Average maps of GSV, AGB and BGB with a pixel size of 100 *m* × 100 m
Type of data	Uint16 GeoTiff files provided (1) in 740 Sentinel-2 tiles i.e. following the applicable UTM zones, for the 20 *m* × 20 m products and (2) as one data file for the 100 *m* × 100 m product in the EPSG:4326 - WGS 84 map projection. Usage notes in pdf format and metadata table in Excel format are provided.
Data collection	The maps were produced with the BIOMASAR approach that inverts a physically-based model relating radar backscatter measurements from the Sentinel-1 and ALOS-2 PALSAR-2 satellite missions and combines several observations to derive an annual estimate of GSV per pixel. The model is supported by forest structural functions trained on spaceborne LiDAR data from the ICESat-1 and ICESat-2 mission and provincial averages of GSV published by National Forest Inventories (NFIs). AGB and BGB were obtained from GSV with allometric equations published in literature. The processing was run on the Forestry TEP platform (https://f-tep.com/).
Data source location	The maps cover 40 European countries, forming a continuous coverage of the western part of the European continent.
Data accessibility	Repository name: Science Data Bank Data identification number: https://doi.org/10.57760/sciencedb.32819 Direct URL to data: https://download.scidb.cn/download?fileId=f4fdeb40a3d67f819df80b7fb2f3d1b0&path=/V2/AGB-2017_20m.zip&fileName=AGB-2017_20m.zip https://download.scidb.cn/download?fileId=d367609adc31c110edc40ed1f3c994bd&path=/V2/AGB-2020_20m.zip&fileName=AGB-2020_20m.zip https://download.scidb.cn/download?fileId=674376aeeba1df67d4fb7bcc99b2f17d&path=/V2/AGB-2021_20m.zip&fileName=AGB-2021_20m.zip
	https://download.scidb.cn/download?fileId=0d3f80f5d9c510bd3d9f791fad20511c&path=/V2/AGB-2023_20m.zip&fileName=AGB-2023_20m.zip https://download.scidb.cn/download?fileId=99ab40561cf95c7ecbf3a7935f4b4578&path=/V2/BGB-2017_20m.zip&fileName=BGB-2017_20m.zip https://download.scidb.cn/download?fileId=07080bc931d8421f50e76d67eecf65da&path=/V2/BGB-2020_20m.zip&fileName=BGB-2020_20m.zip https://download.scidb.cn/download?fileId=35ed9f8c4d97d681e06ef3216df50f1a&path=/V2/BGB-2021_20m.zip&fileName=BGB-2021_20m.zip https://download.scidb.cn/download?fileId=46e97c3ed8f9c0030c65d3d759475660&path=/V2/BGB-2023_20m.zip&fileName=BGB-2023_20m.zip https://download.scidb.cn/download?fileId=56647890659385a61f9bde8208151ecb&path=/V2/GSV-2017_20m.zip&fileName=GSV-2017_20m.zip https://download.scidb.cn/download?fileId=118fff45180aa272735a873d44270db6&path=/V2/GSV-2020_20m.zip&fileName=GSV-2020_20m.zip https://download.scidb.cn/download?fileId=a2a5b969543216de7664d0e0db8d2df8&path=/V2/GSV-2021_20m.zip&fileName=GSV-2021_20m.zip https://download.scidb.cn/download?fileId=2101c621feb3ef3301cd243bfb99abd2&path=/V2/GSV-2023_20m.zip&fileName=GSV-2023_20m.zip https://download.scidb.cn/download?fileId=c6d223ede020c73ea73bc5ae600e5ace&path=/V2/100m_resolution_maps.zip&fileName=100m_resolution_maps.zip https://download.scidb.cn/download?fileId=c8a1abd74e62450859f54343c46e16d4&path=/V2/FCM_paneuropean_biomass_maps_metadata_20260120.xlsx&fileName=FCM_paneuropean_biomass_maps_metadata_20260120.xlsx https://download.scidb.cn/download?fileId=1d0908392f63560abf0635066e900796&path=/V2/FCM_paneuropean_biomass_maps_usage_notes_20260120.pdf&fileName=FCM_paneuropean_biomass_maps_usage_notes_20260120.pdf Instructions for accessing these data: Direct access, registration not required. Full resolution (20 *m* × 20 m) datasets are arranged per year (2017, 2020, 2021 or 2023) and biomass variable (GSV, AGB or BGB). The 100 *m* × 100 m dataset is stored in a single file (all years, all variables). Usage notes are provided as a separate pdf file. A metadata table is included as a separate file.
Related research article	None

## 1 Value of the Data

• Spatially detailed datasets of key forest variables, allowing for precise stratification of timber resources and carbon stocks.
• Repeated map values at any location allow the characterization of biomass dynamics (e.g., abrupt losses, growth, decay).
• The high level of detail allows for accurate computation of (sub)national average values of GSV, AGB and BGB in support of various types of accounting.
• 20 m spatial resolution enables analysis of local level GSV, AGB and BGB distribution
• Comparison with independent reference data shows good relationship with biomass stocks at aggregate levels; pixel-level uncertainties are still significant and validation of biomass changes remains challenging

## 2 Background

Spatially explicit information on forest structure and biomass is needed to meet the monitoring and reporting requirements of several European policies, including e.g., the Nature Restauration [1] the Carbon Removals and Carbon Farming [2] and the Deforestation [3] regulations. Satellites provide the means to produce spatially explicit forest information for large areas in high spatial detail. Timely maps of forest resources are highly needed as European forests experienced substantial changes in recent years due to logging [4] and natural disturbances [5]. The Climate Change Initiative (CCI) Biomass dataset [6] can partially support these requirements, keeping in mind limitations due to the moderate resolution of 1 hectare.

The Forest Carbon Monitoring project (FCM) designed and demonstrated approaches for forest structure and biomass mapping to support a wide range of stakeholders to meet their monitoring and reporting requirements. To obtain a pan-European dataset, the BIOMASAR method was adapted to European forest conditions [7] and generated biomass maps from satellite Synthetic Aperture Radar (SAR) and Light and Detection Ranging (LiDAR) data acquired during the last decade at high spatial resolution (20 *m* × 20 m).

The new mapping methodology complements existing information, e.g., from National Forest Inventories, by enabling annual mapping on forest volume and biomass in high spatial resolution, allowing consistent European wide biomass monitoring in support of EU policy formulation and monitoring.

## 3 Data Description

The dataset consists of maps of forest biomass density, defined either in the form of structural parameters such as Growing Stock Volume (GSV) or in the form of organic dry mass such as Above Ground Biomass (AGB) and Below Ground Biomass (BGB) (Table 1).

**Table 1:** List of the GSV, AGB and BGB map products. The mass unit for AGB and BGB is expressed in tons, this being equivalent to Megagrams (Mg).

File name*	Content	Unit
XXXXX_YYYY_GSV.tif	Growing stock volume	m^3^⋅ha^−1^
XXXXX_YYYY_GSV-SD.tif	Growing stock volume - standard deviation	m^3^⋅ha^−1^
XXXXX_YYYY_AGB.tif	Above ground biomass	tons⋅ha^−1^
XXXXX_YYYY_AGB-SD.tif	Above ground biomass - standard deviation	tons⋅ha^−1^
XXXXX_YYYY_BGB.tif	Below ground biomass	tons⋅ha^−1^
XXXXX_YYYY_BGB-SD.tif	Below ground biomass - standard deviation	tons⋅ha^−1^

*^)^ XXXXX denotes Sentinel-2 tile number and YYYY denotes year.

**Fig. 1:**
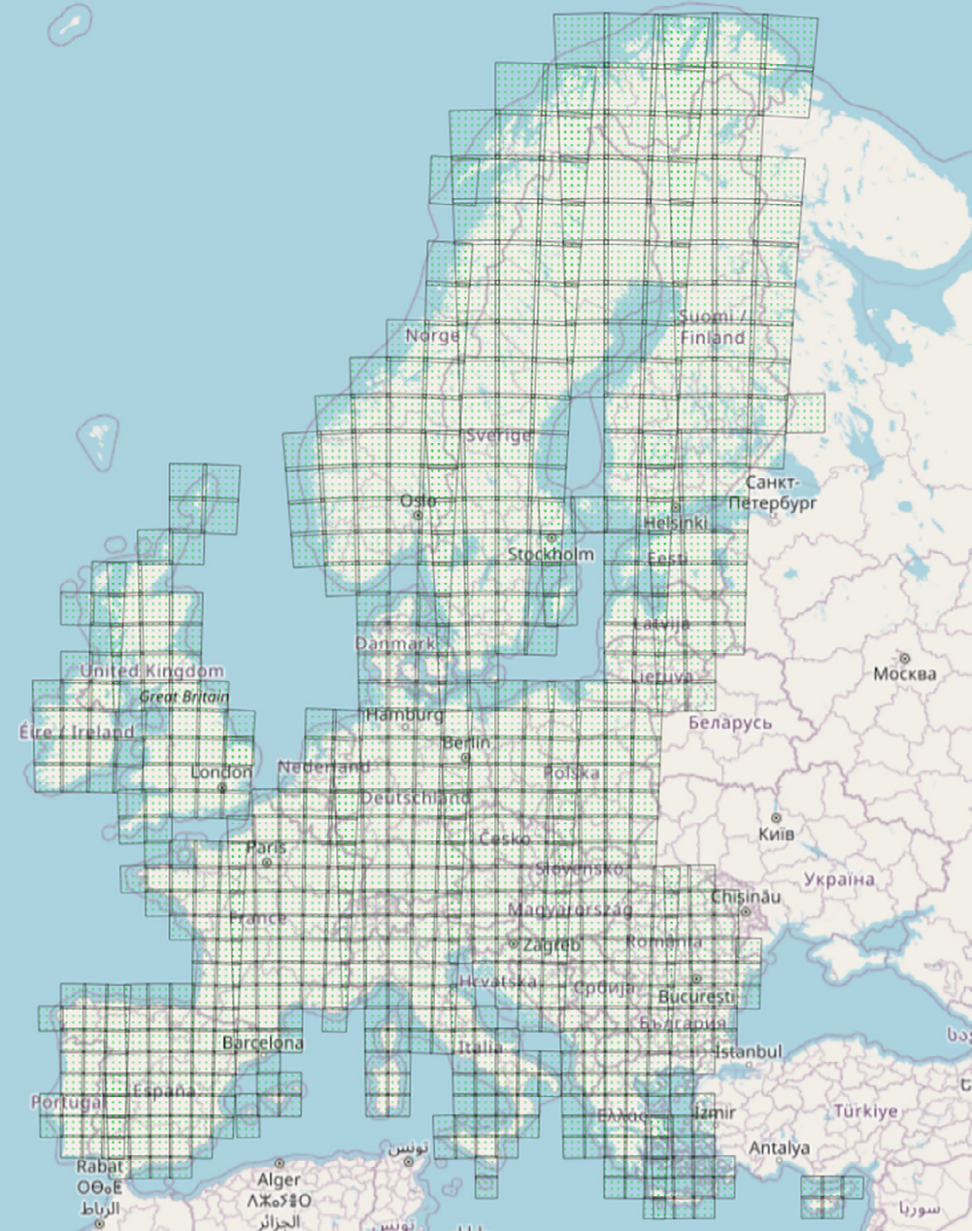
Coverage of the biomass density dataset and geographic distribution of the Sentinel-2 tiling system across Europe.

We aligned our estimation with the approach based on forest field inventory data [[8], [9], [10]] that derives a forest structural variable at first, i.e., GSV. GSV is then used to be the predict carbon-related variables AGB and BGB. The relevance of GSV for the estimation of AGB and BGB is confirmed by the country reports building up the quinquennial Food and Agricultural Organization (FAO) Forest Resources Assessments (FRA). For the FRA of the year 2020 [11], the estimates of AGB and BGB for 33 of the 35 countries within the mapped area (Fig. 1) were derived from an estimate of GSV, using one or several scaling factors.

**Fig. 2:**
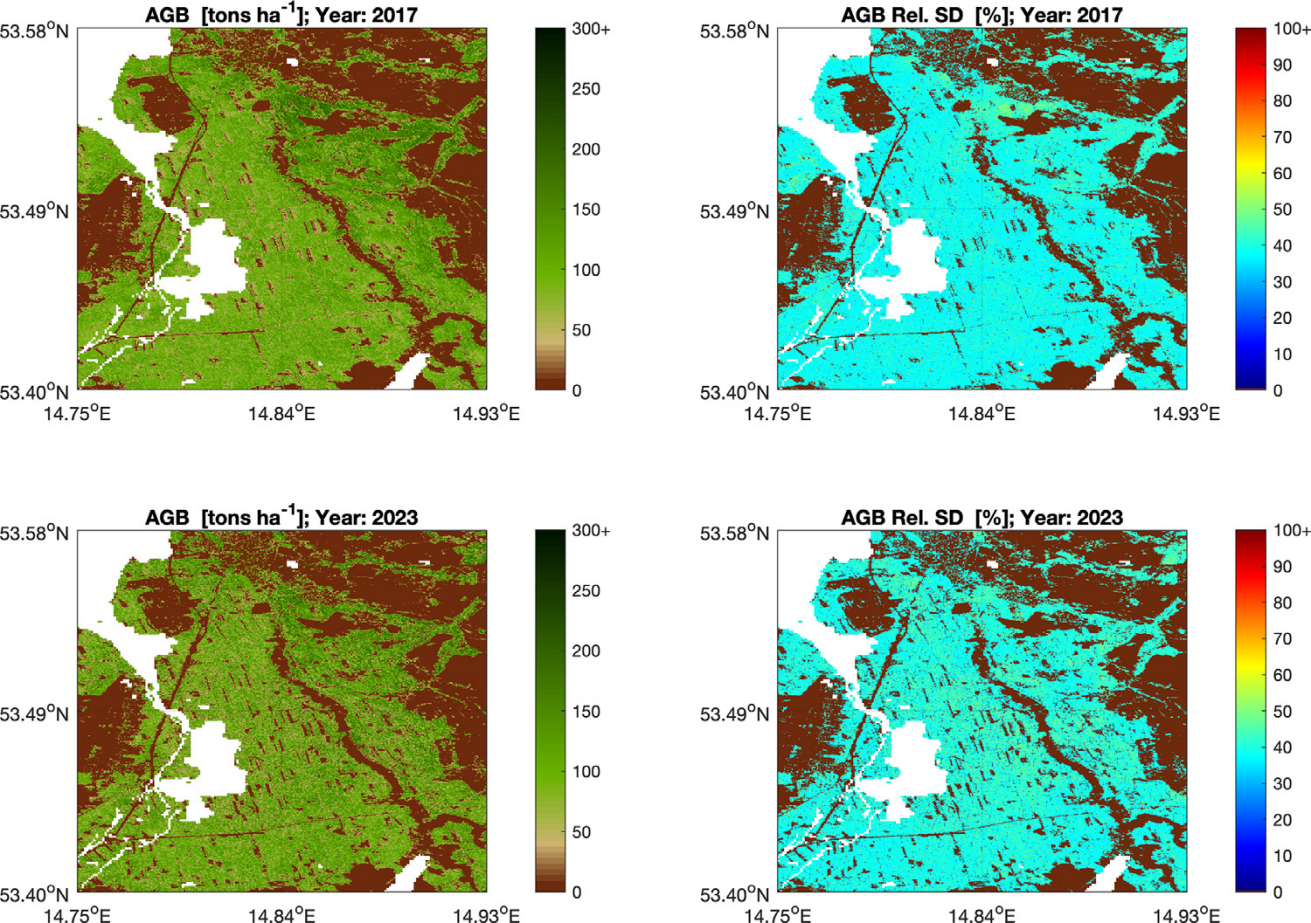
Example of AGB estimates and corresponding SD expressed relative to the AGB value for the years 2017 and 2023 for an approximately 200 km^2^ large area in North Poland.

The maps of GSV, AGB and BGB form a continuous coverage of the European continent (Fig. 1) and are representative of the average biomass conditions in each of the years 2017, 2020, 2021 and 2023. Each estimate of GSV, AGB and BGB is accompanied by a value of the standard deviation (SD). Fig. 2 shows an example of AGB maps and corresponding SDs for the years 2017 and 2023. Loss of biomass is evident in the increase of clear-cut areas between 2017 and 2023. The AGB SD is typically between 40 and 50 % of the estimated value where AGB is high whereas it exceeds 100 % in areas that are unvegetated. The map values are representative of the pixel area, i.e., they do not account for partial or total forest cover within the area of the pixel.

The maps were not masked for forest area because there is not a single universal definition of forest land adopted by potential users of the maps. Users wishing to derive statistics for forest land are advised to apply their forest/non-forest dataset to the data products.

The maps are provided in 740 tiles using the native projection of the Sentinel-2 tile system (Fig. 1), i.e. following the applicable UTM zones. The pixel size of the maps is 20 *m* × 20 m, which corresponds approximately to the native spatial resolution of the satellite image data used to estimate biomass density. Each map is provided as a 16bit Geotiff file.

**Fig. 3:**
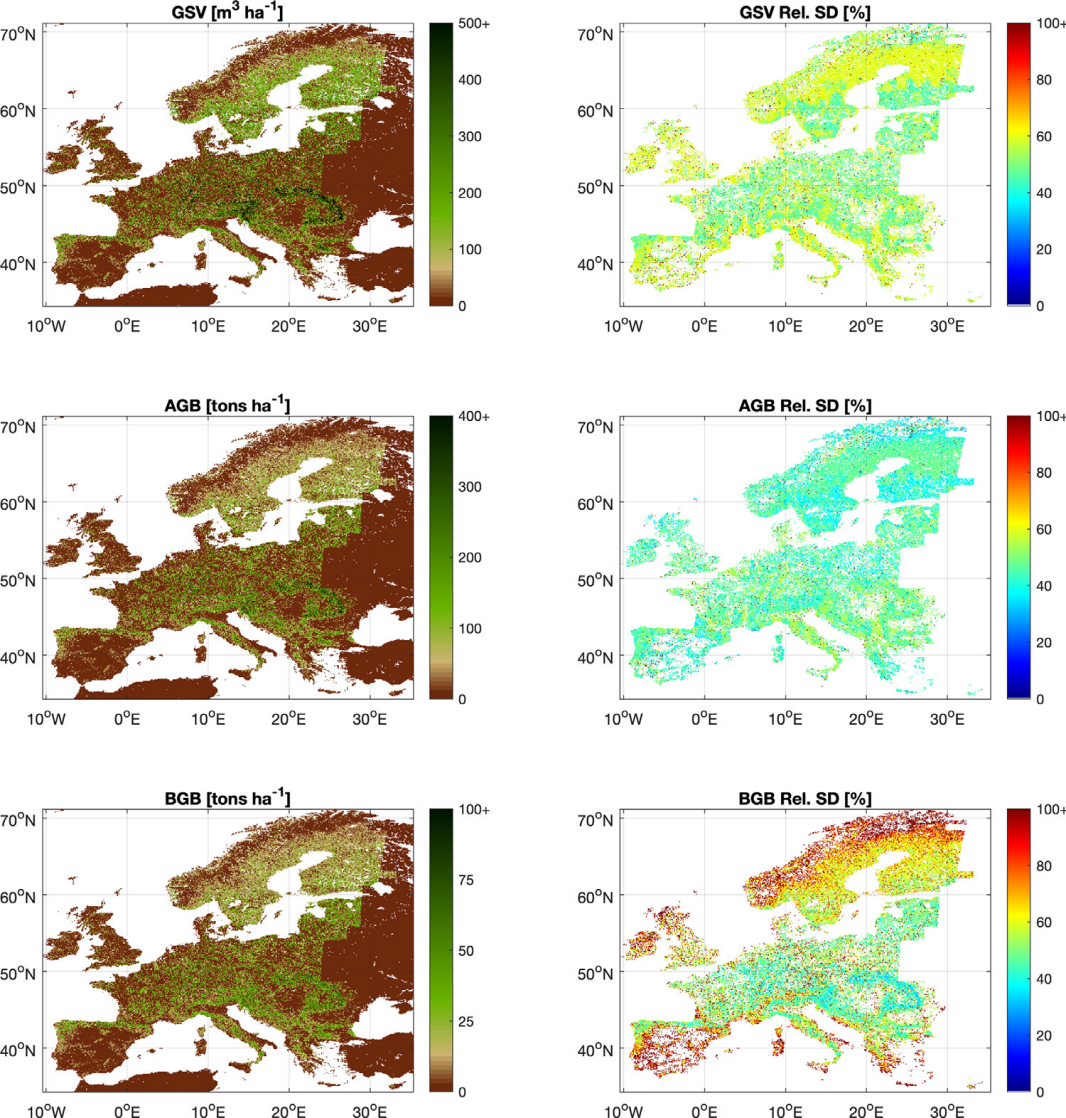
Europe-wide dataset of biomass density maps for the year 2020. The color ramps have been constrained to maximize the color contrast in each panel. For illustrative purposes, the maps in correspondence of open water surfaces are made transparent. Full transparency was also used in the maps displaying the relative SD in correspondence of pixels with AGB = 0.

In addition to the full resolution files, also single files covering the entire area mapped are provided with a pixel size of 100 *m* × 100 m. These files are named as FCM_Europe_demo_<GSV/AGB/BGB (-SD)>.tif, using the same variable acronyms as in the full resolution files. The European wide 100 m resolution product is in the EPSG:4326 - WGS 84 map projection (Fig. 3). The product was generated by spatially averaging the full resolution map and resampling to the geographic projection.

## 4 Experimental Design, Materials and Methods

### 4.1 Satellite datasets

The map products were derived from satellite Synthetic Radar Aperture (SAR) image data acquired by the Sentinel-1 SAR and the Advanced Land Observing Satellite 2 (ALOS-2) Phased Array l-band Synthetic Aperture Radar-2 (PALSAR-2) instruments because of their sensitivity to forest structure.

Sentinel-1 (S1) is a satellite mission operated by the European Commission in the Copernicus framework since 2014 and consists of four identical satellites (1A, 1B, 1C and 1D) each operating a C-band SAR (wavelength of 5.6 cm). For this work, images acquired by the 1A and 1B units were considered. The 1C and 1D units were launched in 2025. Each satellite unit has a 12-day repeat-pass interval. When combined, two satellites provide for a six-day repeat coverage and even more frequent observations when considering the overlap between adjacent orbits, in particular at high latitudes. Over Europe, the SAR is operated in dual-polarization mode (Vertical-Vertical, VV, and Vertical-Horizontal, VH) and each satellite acquires continuously along both ascending and descending orbital tracks. Combined, Sentinel-1A and 1B acquired a total of about 60,000 scenes and on average 60 observations per pixel and per year. Given the high temporal correlation of backscatter observations, the Sentinel-1B dataset was redundant as it would have not improved the biomass estimation. Hence, we used only Sentinel-1A images, which resulted in approximately 30,000 SAR images per year.

ALOS-2 is a satellite mission operated by the Japan Aerospace Exploration Agency (JAXA) since 2014 and carries the longer wavelength (23.5 cm) PALSAR-2 L-band SAR instrument. For this work, ALOS-2 data acquired in dual polarization (HH and HV) stripmap mode were processed and provided by JAXA through a dedicated CCI collaboration effort. All data acquired over Europe during each of the four years of the biomass mapping were used. The ALOS-2 data were provided in the form of ca. 300 km long strips of detected data (i.e., backscatter amplitude only) at 25 m pixel spacing.

The SAR datasets consisted of images of the radar backscatter and were provided either in ground-range geometry (Sentinel-1) or in slant-range geometry (PALSAR-2). The images were then pre-processed to obtain stacks of terrain geocoded, radiometrically calibrated, speckle-filtered and co-registered images. Pre-processing consisted of:

1. multi-looking in range and azimuth to obtain pixels with 20 m x 20 m ground pixel posting;
2. compensation for the noise equivalent sigma nought (NESZ) (Sentinel-1 only);
3. updating of orbit state vectors with precision orbit vectors provided by ESA within 20 days past the image acquisition^1^ (Sentinel-1 only);
4. radiometric terrain correction accounting for varying pixel scattering areas dependent on topography to produce “terrain-flattened” backscatter intensity images [12];
5. geocoding and orthorectification based on the Copernicus 1-arcsecond Digital Elevation Model (DEM) to the target UTM map grid with 20 *m* × 20 m pixel size [13].

**Fig. 4:**
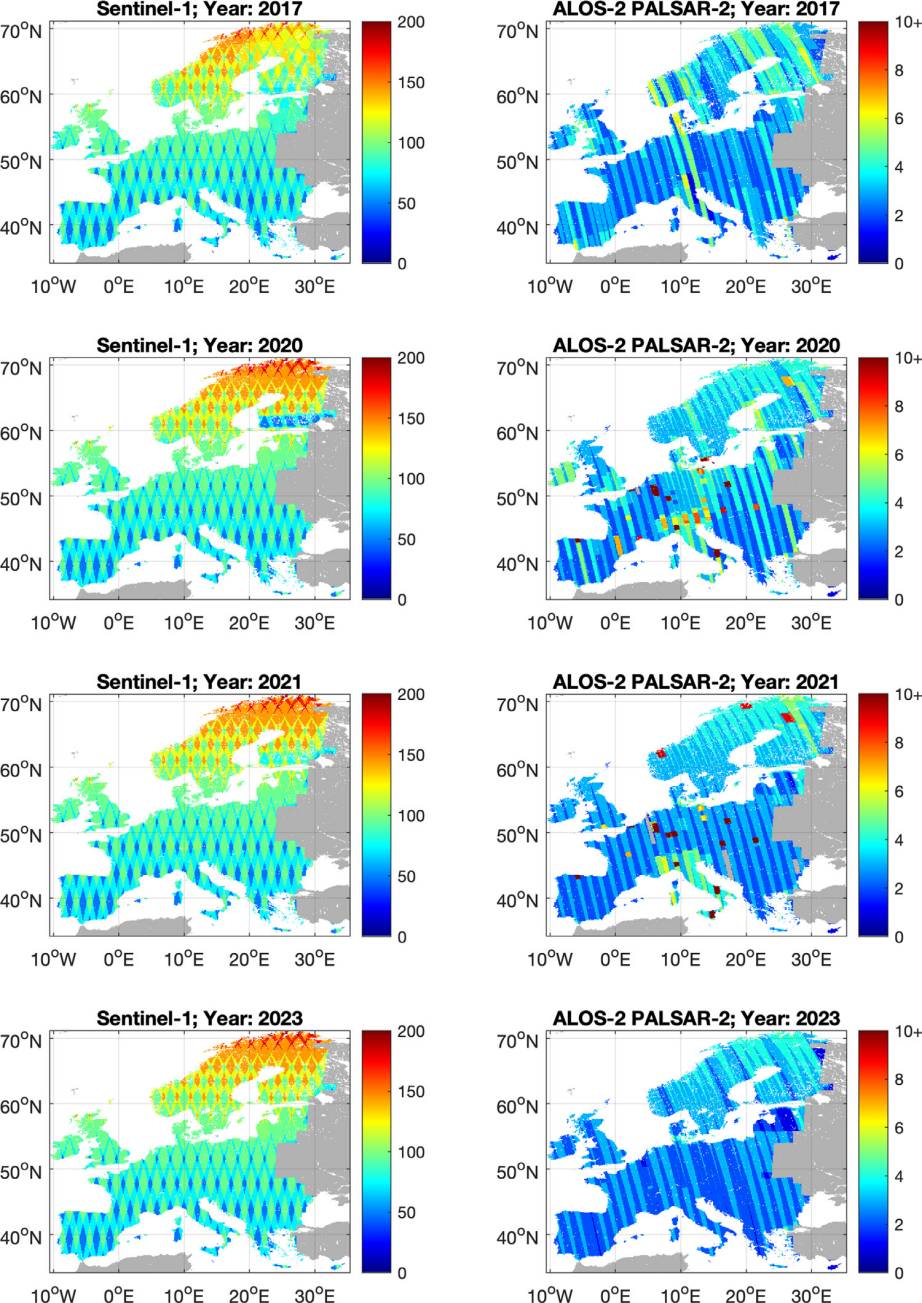
Number of SAR backscatter observations per pixel, per year and SAR satellite.

All geocoded images were resampled to the same MGRS/UTM tiling grid to ESA’s Sentinel-2 dataset to allow for a joint use/inter-comparison of data products based on radar and optical sensors. Fig. 4 shows the number of observations per pixel and satellite for each of the four mapped years. Sentinel-1 observations were regularly acquired throughout each year whereas the frequency of ALOS-2 observations decreased in time and was often restricted to one image per year.

### 4.2 Estimation of forest biomass density

Estimation of forest biomass density requires observations of both the horizontal (i.e., tree density) and vertical (i.e., height) properties of a forest. To estimate the mass, tree form factors and wood densities are additionally needed. Remotely sensed data from space do not offer such a variety of observations. Therefore, biomass can only be inferred by means of mathematical models, which are tailored to adapt to remotely sensing data available with the aid of reference biomass data from ground surveys. This aspect becomes even more crucial when available measurements have limited sensitivity to biomass, which is the case for satellite missions with imaging instruments currently in operation.

Because of the unavailability of spatially dense datasets of reference biomass measurements across Europe, biomass was estimated with the BIOMASAR approach [14]. This physics-aware method relates SAR and LiDAR observations to the biomass variable of interest, i.e., GSV or AGB, and does not require plot-based measurements for training the inversion model.

Here, we used the set up published in [7], which generated per-pixel estimates of GSV from Sentinel-1 and ALOS-2 PALSAR-2 observations. GSV was retrieved with the radar backscatter model in Eq. (1) which includes explicit information on forest structural conditions. The model integrates structural functions that relate horizontal and vertical structural parameters (i.e., canopy density and canopy height) to GSV

(1) $$\sigma_{for}^{0}=\sigma_{gr}^{0}\left(e^{-q{\left(aV\right)}^{b}}+e^{-\alpha {\left(aV\right)}^{b}}-e^{-\left(q+\alpha \right){\left(aV\right)}^{b}}\right)+\sigma_{veg}^{0}\left(1-e^{-q{\left(aV\right)}^{b}}-e^{-\alpha {\left(aV\right)}^{b}}+e^{-\left(q+\alpha \right){\left(aV\right)}^{b}}\right)$$

In Eq. (1), the backscatter from the forest, *σ°_for_*, was related to the backscatter from the ground floor, *σ^0^_gr_*, and the vegetation layer, *σ^0^_veg_*, with respect to the relative contribution to the total backscatter by each term. The transmissivity terms in brackets are related to the coefficient *α* expressing the two-way attenuation per meter through a tree canopy and the depth of the attenuating layer, which was assumed to correspond to the canopy height. The coefficient *α* was equal to 2dB/m at C-band and 0.5 dB/m at l-band in line with known values from literature [7]. In Eq. (1), the coefficient *q* is part of a structural function that expresses canopy density as a function of canopy height described in [7]. Canopy height is finally replaced with GSV (*V*) using a second structural function in the form of a power-law model with coefficients *a* and *b* described in [7].

The rationale behind the estimation of the coefficients *q, a* and *b* was presented in [7] and lead to spatially explicit datasets. Calibration of Eq. (1) consisted of an estimation of the model parameters *σ^0^_gr_* and *σ^0^_veg_* for each Sentinel-1 and ALOS-2 PALSAR-2 image. To adapt to local forest structural conditions, the calibration was undertaken separately for each Sentinel-2 tile. In [7], two approaches to obtain these estimates were presented.

1. Select pixels in correspondence with sparse and dense tree cover using to a map of tree cover density as reference and calculate the mean values of the observed backscatter in such areas. The estimate of *σ^0^_gr_* corresponds to the mean value of the backscatter for sparse tree cover (i.e., < 30 %). The estimate of *σ^0^_veg_* is obtained from the mean value of the backscatter for dense forest cover (i.e., > 0.75⋅maximum tree cover, as in [14]) with a compensation for residual backscatter from the ground through gaps [7].
2. Express Eq. (1) as a function of tree cover density as described in [7] and regress the backscatter to corresponding value of tree cover density. The estimate of *σ^0^_veg_* was obtained by compensating the regression-based value with the same procedure as in point 1.

Both approaches were tested on the C- and l-band dataset by inverting the model in Eq. (1) and checking whether the retrieved GSV were affected by systematic biases. The most reliable solution consisted of applying the first approach to calibrate the model at l-band and the second approach to calibrate the model at C-band. However, the estimates of *σ^0^_gr_* at C-band were occasionally quite uncertain because of the very large range of values of the backscatter for sparsely vegetated surfaces. To overcome this issue, we implemented a cruder estimation developed for global biomass mapping [6], which consisted of setting *σ^0^_gr_* equal to the 25th percentile of backscatter values from pixels selected as sparsely vegetated.

Once all parameters in Eq. (1) were set, GSV was retrieved for each SAR image. To improve the accuracy, the individual estimates of GSV from the same set of SAR images were first combined with a weighted average following [7].

(2) $$GSV_{mt}=\frac{\sum_{i=1}^{N}w_{i}GSV_{i}}{\sum_{i=1}^{N}w_{i}}$$

The weights in Eq. (2) were defined as the difference between the backscatter parameters of the WCM, *w_i_=σ^0^_veg_ − σ^0^_gr_*.

The final estimate of GSV, *GSV_fin_*, was then obtained from the C- and l-band GSV estimates, *GSV_mt,C_* and *GSV_mt,L_*, with a second weighting described in Eq. (3) and justified in [7].

(3) $$GSV_{fin}=w\left(L\right)\cdot GSV_{mt,L}+w\left(C\right)\cdot GSV_{mt,C}$$

The weights *w(L)* and *w(C)* were expressed as a function of the expected transmissivity corresponding to the estimated GSV, the number of SAR observations at C- and l-band and the terrain slope. It should be noted that for estimation of GSV or AGB in forested areas in particular, the longer l-band wavelength (23.5 cm) of ALOS-2 PALSAR-2 was of critical importance.

To estimate AGB and BGB, we used allometric equations published by [15], which were developed on extensive measurements of forest variables from European forests. The allometric equations were stratified by leaf type, i.e., broadleaves and conifers, the latter category including both needleleaf deciduous and needleleaf evergreen forests. For the stratification of the landscape by leaf type, we used Copernicus High Resolution Layer of Dominant Leaf Type [16], which was resampled to the pixel size of the biomass maps with nearest neighbor interpolation.

To estimate AGB from GSV, GSV was first converted to stem biomass, SB, with an estimate of the wood density, WD (unit: g/cm^3^). For the wood density, average values per leaf type reported by Thurner et al. (2014) were used.

(4) SB=GSV·WD

SB was used to estimate the biomass density in branches, BB and foliage, FB, and roots, RB. We used the leaf-type specific power-law functions published in [15]. AGB was obtained from the sum of the stem, branch, and foliage biomass density estimates. BGB was equal to the estimate of the roots’ biomass, *RB*.

(5) AGB=SB+BB+FB

GSV, AGB and BGB were estimated for each pixel regardless of the land cover type. However, the WorldCover dataset of 2020 [17] was used in post-processing to remap GSV, AGB and BGB to 0 tons⋅ha^−1^ in correspondence of pixels labelled as built-up areas or permanent water bodies. The moderate or high backscatter values characterizing such areas converted to unrealistically high values of biomass. To match the pixel size of the biomass density maps, the WorldCover dataset underwent majority resampling.

### 4.3 Uncertainty of the forest biomass density estimates

Each estimate of GSV was accompanied by a value of its standard deviation, which was obtained by propagating errors of the individual satellite observation and the model parameters [7].

The SD of a GSV prediction from a measurement of the backscatter was obtained by perturbing the measurement and the prediction model parameters (*σ^0^_gr_, σ^0^_veg_, q, a* and *b*) using the SD values obtained during their estimation. The SD of the prediction was then defined as the standard deviation of the vector of perturbed GSVs obtained by repeating the perturbation *N* = 100 times. The variance of the GSV prediction from Eq. (2) was then the sum of a variance component and a covariance component that accounts for the temporal correlation of prediction errors at a given pixel.

(6) $$\delta {\left(GSV_{mt}\right)}^{2}=\sum_{i=1}^{N}w_{i}^{2}\cdot \delta {\left(GSV_{i}\right)}^{2}+2\cdot \sum_{i=1}^{N-1}\sum_{j=i+1}^{N}w_{i}\cdot w_{j}\cdot Cov\left(GSV_{i},GSV_{j}\right)$$

where

(7) $$Cov\left(GSV_{i},GSV_{j}\right)=\delta GSV_{i}\cdot \delta GSV_{j}\cdot r_{ij}$$

In Eq. (6), the variance component was modelled as a linear combination of the variances associated with the individual stem volume estimates *δ(Vi)^2^*, where *w_i_^2^* is the weight introduced in Eq. (2), following [18]. The covariance component was expressed in a similar manner where individual error co-variances were weighted. The error covariance in Eq. (6) was obtained from the pairwise standard deviation of GSV estimates from image *i* and image *j* and the corresponding correlation of errors, *r_ij_*. Here we used a single global constant for *r_ij_* equal to 0.52, based on the analysis reported in [6].

The variance of the GSV estimate was from the combination of the BIOMASAR-C and -L AGB estimates was finally obtained as in [6].

(8) $$\delta {\left(GSV_{fin}\right)}^{2}=w^{2}\left(L\right)\delta {\left(GSV_{mt,L}\right)}^{2}+w^{2}\left(C\right)\delta {\left(GSV_{mt,C}\right)}^{2}$$

The variance of the AGB was obtained by adding the variances of the stem, branch and foliage biomass components (Eq. (9)).

(9) $$\delta {\left(AGB\right)}^{2}=\delta {\left(SB\right)}^{2}+\delta {\left(BB\right)}^{2}+\delta {\left(FB\right)}^{2}$$

The variances of the branch and the foliage biomass were modelled as in [15]. The variance of the stem biomass prediction was obtained with Eq. (10) and accounted for the variance of the stem volume prediction from Eq. (8) and of the wood density. The variance of the wood density, *δ(WD)^2^*, was modelled using the second order polynomials developed for European forests as in [15].

(10) $$\delta {\left(SB\right)}^{2}={\left(WD\right)}^{2}\cdot \delta {\left(GSV_{fin}\right)}^{2}+{\left(GSV_{fin}\right)}^{2}\cdot \delta {\left(WD\right)}^{2}$$

The variance of the BGB was equal to the variance of the root biomass, which was modelled as in [15].

### 4.4 Validation of the biomass density maps

Rigorous validation of large-scale of biomass estimates is hindered by the unavailability of wall-to-wall, temporally and spatially harmonized measurements. Assessments of wall-to-wall maps typically rely on opportunistic datasets gathered from various sources [19]. Measurements taken at field inventory plots as part of research networks, provincial surveys or commercial activities, may support a detailed assessment of the map at the local scale but would not provide indications about the overall reliability of the map itself. Nation-wide plot-based datasets collected by European NFIs would help in this direction. Nonetheless, inventory protocols differ from country to country, and access to the data is mostly restricted. In addition, a direct comparison of plot-based values with corresponding pixel values is not informative of the accuracy of a map [19]. The similarity of information provided by plots and maps increases by spatial averaging. In this respect, a comparison of grid-cell based average values from plots and pixels provides a more correct assessment of the map’s accuracy. The accuracy of the individual pixel values cannot be quantified though.

The availability of GSV and AGB field inventory data shaped our validation activities. The BGB maps could not be validated because we lacked reference measurements. GSV data were available for four sites in Catalonia, North Finland, South Finland and Romania [7], and in the form of average values at administrative level gathered from reports published by NFIs for 26 countries [18]. It is noted that NFIs typically do not report AGB statistics so that a similar analysis for AGB could not be undertaken. The accuracy assessment for AGB was instead undertaken in line with the new CEOS Land Product Validation protocol for biomass from space calibration and validation [20] thanks to the availability of an extensive dataset of plot data from NFIs of Spain, Ireland, The Netherlands, Belgium, Italy, Croatia and Sweden. These datasets are part of a global collection of forest AGB values that were gathered from a variety of surveys undertaken by NFIs and research networks [21]. None of these data were used for calibration of the inversion models so that they are fully independent from the production process. The implications of such an opportunistic collection of reference measurements is discussed in the next section.

**Fig. 5:**
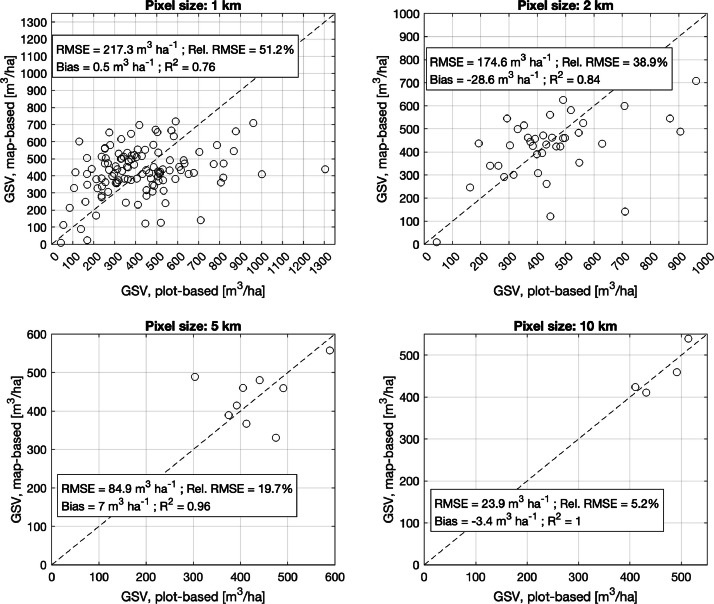
Scatter plots comparing average values of GSV from the map product and from plot inventory measurements for different spatial aggregation levels between 1 and 10 km. The retrieval statistics include the root mean square error (*RMSE*), the RMSE value relative to the mean plot-based GSV value, the difference between map-based and plot-based average values (bias) and the coefficient of determination, *R^2^*.

The approach that estimates GSV was already validated at four sites in Europe with measurements from forest field data collected at inventory plots having a size comparable to the pixels of the biomass density maps [7]. The spatial distribution of GSV was captured at the maps’ full resolution (20 m); however, the uncertainty was remarkable (root mean square error relative to the mean GSV larger than 50 %). Here, we extend that validation exercise by assessing the effect of spatial averaging of the maps to coarser pixel sizes. The accuracy of the map-based averages when compared to corresponding average values of the plot-based GSVs increased with the size of the averaging window. Fig. 5 shows an example for the site located in Romania where plot-based and map-based average values of GSV are compared at different aggregation levels. The variance decreased with increasing spatial aggregation as also confirmed by the R^2^ value, which increased from 0.15 at full resolution (not shown here) to 0.76 for the 1 km pixel size and close to 1 for pixel sizes larger than 5 km. The main drawback of spatial averaging is that the range of GSV values is considerably reduced.

To validate the AGB maps, two temporally matched subsets were prepared. The 2020 subset was used to validate the 2020 AGB map, whereas the 2015 subset was paired with the 2017 AGB map. The dataset used to validate the 2017 map did not include measurements from the same year. As a compromise between data availability and temporal proximity, we selected 2015 as the reference year. This strategy limited growth-rate mismatch while guaranteeing an adequate sample size for error calculation.

**Fig. 6:**
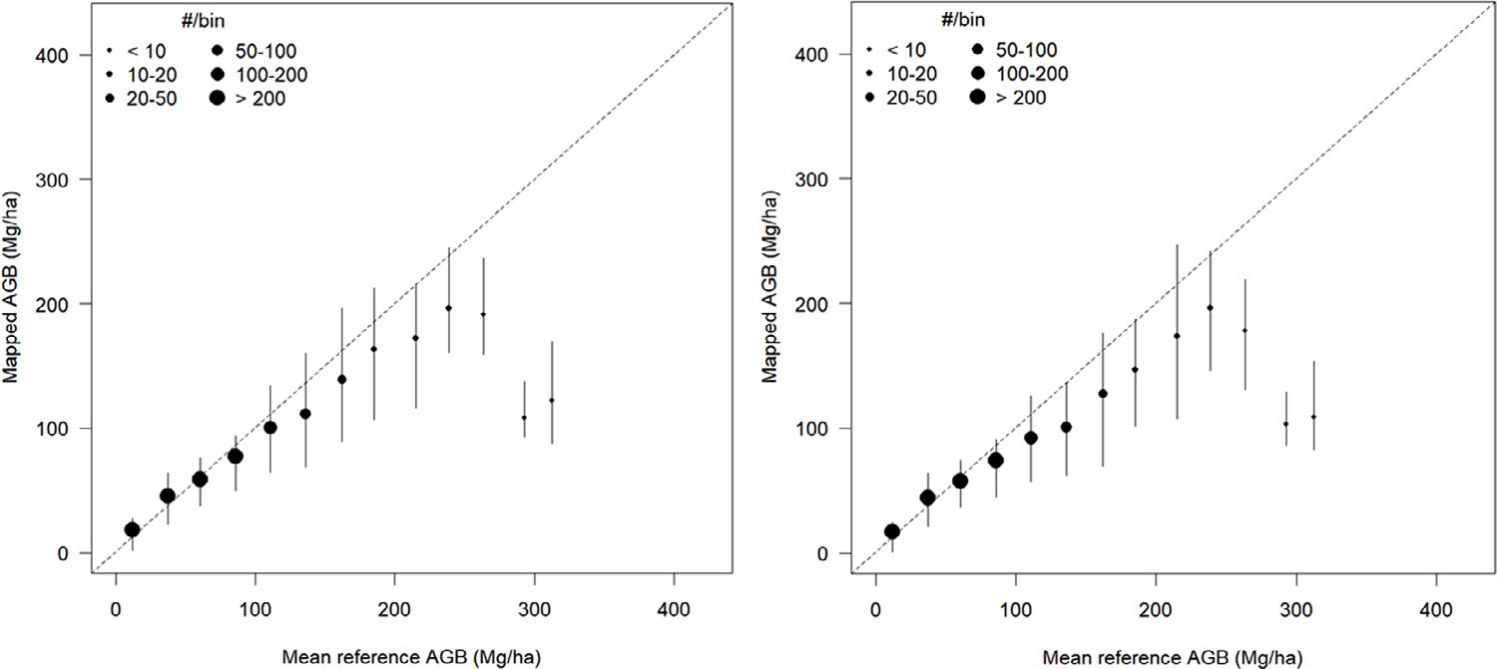
Comparison of plot-based (i.e., reference) and mapped average AGB at grid cell level for 2017 (left panel) and 2020 (right panel). In each panel, the comparison is visualized with the median (filled circles) and the interquartile range (vertical bars) of map-based AGBs per bin of the AGB reference values. The size of the filled circles is proportional to the number of data points in each bin, which is visualized with the color ramp on the right hand-side of each panel.

In accordance with the validation scheme used for global biomass data products, the validation was undertaken at the level of average values from plots and map-based values using a 10×10 km^2^ grid [19]. Grid cells containing less than five field plots were, however, excluded, in accordance with the ``minimum-plots'' quality flag. The filtered locations formed a dense corridor across continental Europe, with highest concentrations in France, Germany, Poland and the Czech Republic as well as across the Baltic–Scandinavian belt. Comparison of plot-based and map-based grid cell averages (Fig. 6) shows strong inter-annual consistency with AGBs in close agreement up to about 250 tons⋅ha^−1^. Systematic underestimation emerges at around 300 tons⋅ha^−1^, which was caused by an incorrect constraint on the maximum retrievable biomass.

**Fig. 7:**
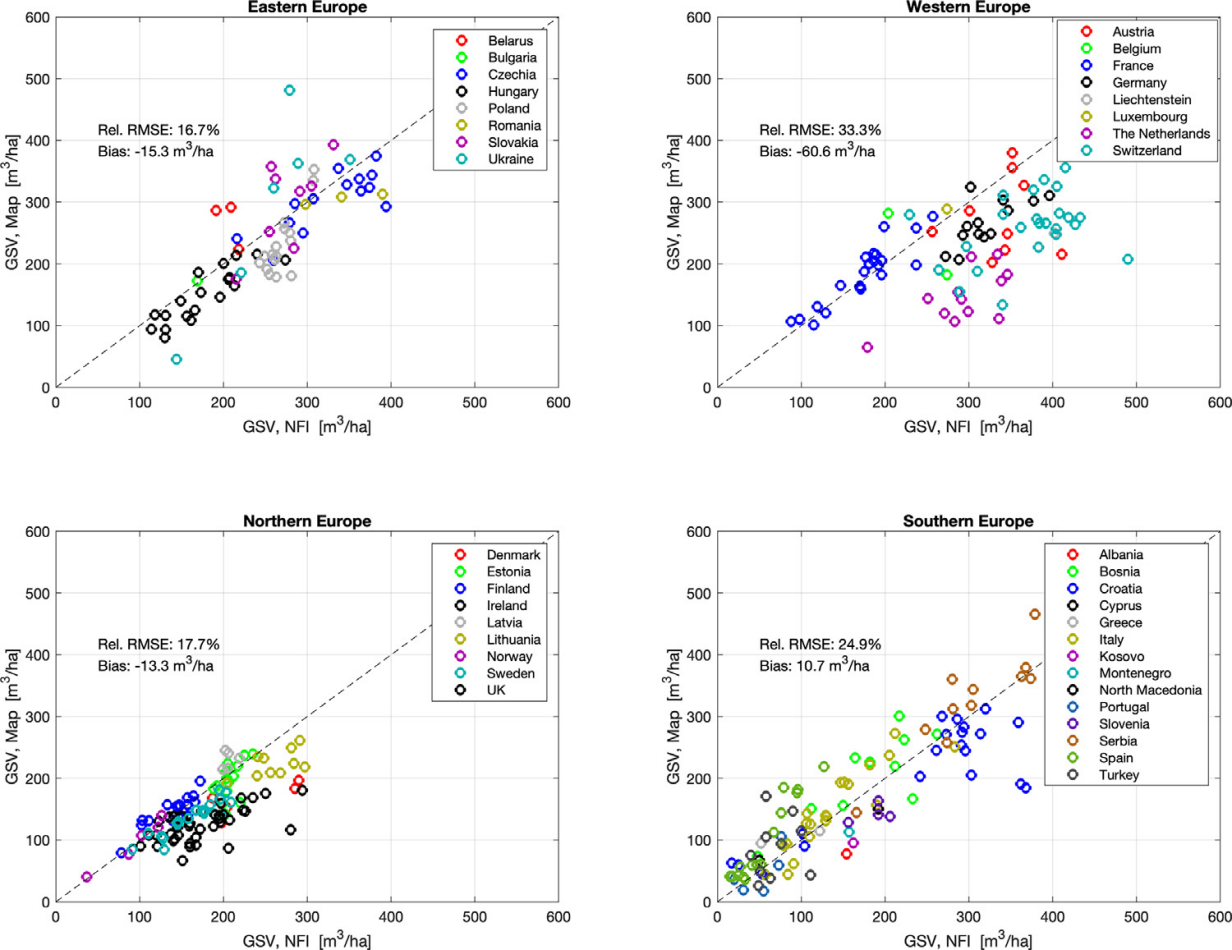
Comparison of GSV averages at subnational level from the map product of 2017 with values published by NFIs. Administrative units are grouped by country. Countries have been into four panels according to their geographic location for better interpretability. Retrieval statistics include the relative RMSE and the bias value.

The analysis at sub-national level compared averages from the map product of GSV at the level of individual provinces with values reported by European NFIs in their periodic reports on forest resources (Fig. 7). The Copernicus High Resolution Layer was used to mask out non-forest areas and produce map-based average values comparable to the numbers obtained by the NFIs. Even if the GSV statistics by the NFIs were based on a different definition of forest area and were not always matching in time with the map-based values, this comparison was indicative of the quality of the FCM data product. For Nordic and Mediterranean countries, the agreement was strong. For countries in Central Europe (i.e., Germany, Poland, Czech Republic, Hungary), the map underestimated GSV. Here, we identified an issue with calibration of the retrieval model. The Copernicus High Resolution Layer was used as auxiliary dataset for tree cover density to calibrate Eq. (1). In correspondence of the class “pastures”, this dataset did not report tree cover densities with the effect of reducing substantially the number of pixels on which the model could have been calibrated. As a consequence. the prediction model was biased. Strong topography caused underestimation in Switzerland. Strong land fragmentation and country-specific forest definitions explained the discrepancies in the United Kingdom, Ireland and the Netherlands.

## Limitations

Map values refer to the dry biomass stored in live, woody vegetation. However, the maps were derived from radar backscatter observations, which are sensitive to size, shape and water content, so that values larger than zero might have occurred in correspondence of dead standing wood or non-woody vegetation. In case of layered vegetation, the biomass of the understory is furthermore not accounted for because SAR data used to estimate biomass did not contain sufficient signal scattered from layers below the top canopy.

Because of the large SD of the estimates and local systematic biases, the individual pixel values are quite uncertain. In addition, biomass values in areas affected by changes within a given year must be disregarded because they may not be representative of the true biomass either before or after the change.

Due to the opportunistic nature of the datasets available to assess the biomass maps, our validation does not provide an exhaustive picture of their accuracy. While the spatial distribution of the biomass appears to have been captured correctly, the maps show some regional biases whereas more local biases could not be quantified. Map users that intend to use a portion of the biomass maps are strongly encouraged to perform an own assessment and interact with the map producers to get a deeper understanding of the map errors.

**Fig. 8:**
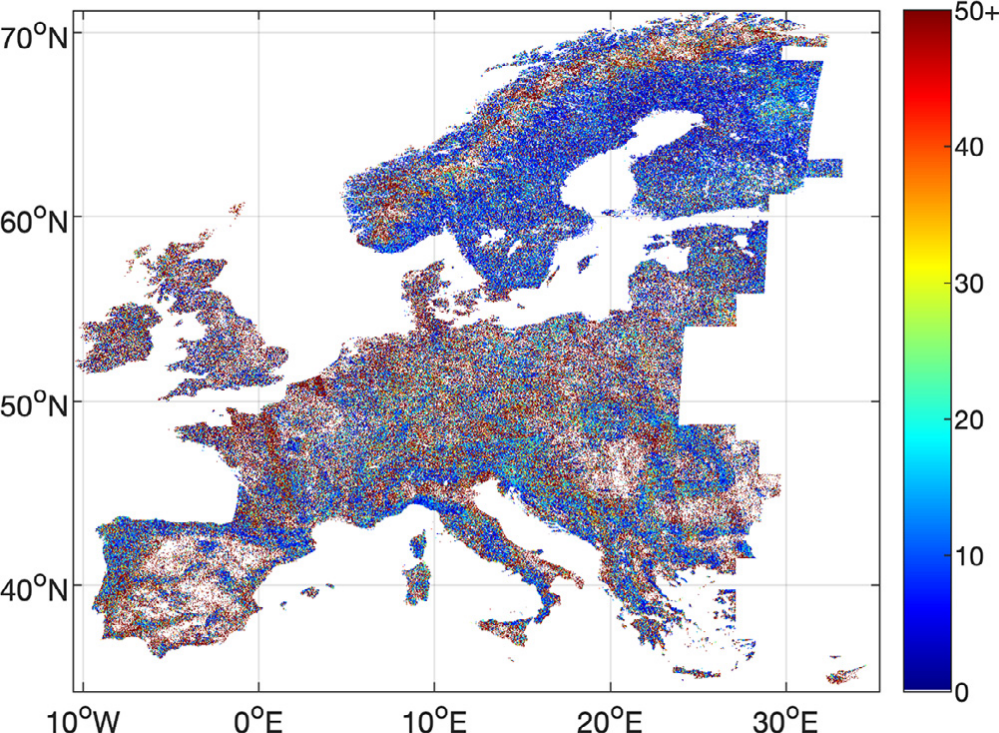
Coefficient of variation expressed in % of the GSV value for the four GSV maps.

Fluctuations of the map values from one year to the next may occur due to the inter-annual variability of the number of radar observations (Fig. 8). The coefficient of variation of the GSV relative to the GSV estimate in Fig. 8 shows banding, in particular in regions where the frequency of ALOS-2 PALSAR-2 observations was low and variable in time. Therefore, trends in the time series of the maps may need to be critically investigated.

## Ethics Statement

The authors have read and follow the ethical requirements for publication in Data in Brief and confirm that the current work does not involve human subjects, animal experiments, or any data collected from social media platforms.

## CRediT Author Statement

**Maurizio Santoro:** Conceptualization, Methodology, Software, Formal analysis, Investigation, Writing, Visualization, Supervision; **Oliver Cartus**: Methodology, Software, Data curation, Writing; **Arnan Araza**: Validation, Formal analysis, Data curation, Visualization; **Martin Herold**: Validation, Investigation, Writing – Review & Editing; **Jukka Miettinen**: Conceptualization, Writing, Visualization, Supervision, Project administration, Funding acquisition; **Ake Rosenqvist**: Resources, Writing – Review & Editing; **Kazufumi Kobayashi**: Resources, Data curation, Writing – Review & Editing; **Takeo Tadono**: Resources, Writing – Review & Editing; **Frank Martin Seifert**: Writing – Review & Editing, Supervision.

## Data Availability

https://www.scidb.cn/enFCM biomass maps for Europe (Original data).

## Footnotes

1 https://qc.sentinel1.eo.esa.int/aux_poeorb/.
